# The Palatal Elevation Technique (PET) for Intra-alveolar Extraction of Grossly Decayed Maxillary Third Molars

**DOI:** 10.7759/cureus.46127

**Published:** 2023-09-28

**Authors:** Rakshit V Sinai Khandeparker, Rahul D Kamat, Omkar A Shetye, Pooja Mandrekar, Sayali K Desai, Vikas Dhupar

**Affiliations:** 1 Oral and Maxillofacial Surgery, Goa Dental College and Hospital, Bambolim, IND

**Keywords:** luxation, fulcrum, palatal bone, elevator, maxillary third molar

## Abstract

Intra-alveolar extraction of maxillary third molars always poses a challenge to dental practitioners owing to limited accessibility and minimal space for dental forceps application. Dental elevators facilitate the extraction of such teeth. In the traditional technique as described in the literature, the elevator is always introduced from the mesiobuccal aspect of the tooth to engage the space between the interdental bone and the offending tooth to use it as a fulcrum. However, certain situations prevent proper application of the elevator from the buccal aspect of the offending tooth to bring about luxation. One such situation is a grossly decayed third molar tooth, especially from the mesiobuccal aspect with destruction of the tooth substance extending below the cementoenamel junction. Another such situation is observed in patients presenting with thick and inextensible cheeks but a good interincisal opening. In either situation, it becomes very challenging to achieve a good purchase for luxation of the offending third molar. The authors have therefore described a modified technique of tooth elevation, the palatal elevation technique (PET), using the palatal bone instead of the buccal bone as the fulcrum which was observed to be effective in such situations. In the authors' view, PET is simple and quick and can effectively be employed as an alternative to the traditional technique of tooth elevation in all cases that require an intra-alveolar extraction of maxillary third molars.

## Introduction

Dental elevators are instruments used to impart luxation forces to the tooth which severe the periodontal ligament which connects the tooth with the surrounding alveolar bone along the surface of the tooth. This expansion increases the degree of freedom of tooth movement inside the socket [1-3]. The combination of all these effects results in the extraction of the tooth or facilitates systematic forceps extraction of the tooth roots. Luxation is also seen to bring about the expansion of the space between the tooth roots and the surrounding alveolar bone by severing the periodontal ligament and pushing the roots away from the alveolar bone surface [4]. The maxillary third molars pose a challenge to the dental practitioner due to limited access and insufficient space for proper forceps application. The posterior position in the oral cavity impedes maintaining the tooth clean, especially on the buccal aspect, making this area a favored location for caries. In some instances, the mesiobuccal crown structure is seen to be completely destroyed by caries extending below the cervical margin and below the bone level [4]. This eliminates the possibility of correctly placing the dental elevator from the mesiobuccal aspect of the offending tooth for the purpose of bringing about tooth luxation. Furthermore, on the buccal aspect of the third molar, the bone is seen to be flat which makes application of forceps also difficult in this region in such a situation. The weakened tooth structure can result in a fracture of the tooth if subjected to dental forcep application and tooth delivery without prior tooth luxation using elevators. A fractured tooth will then necessitate usage of either chisel and mallet or bur and handpiece which can add to the surgical complexity. This can significantly affect the patient’s quality of life particularly during the first two days after extraction [5]. It is usually observed based on the authors' experience that in such teeth, the palatal crown structure above the cementoenamel junction is intact and the strong palatal bone on the mesial aspect of the tooth offers a good purchase point for the elevator for tooth luxation. The authors have therefore described a modified technique of tooth elevation using the palatal bone instead of the buccal bone as a fulcrum which can effectively be used in such a situation. In the authors' opinion, the novel technique offers a simple and quick solution for the extraction of grossly decayed maxillary third molars without resorting to more advanced exodontia techniques of tooth removal.

## Technical report

All the patients who underwent extraction using the modified technique of tooth elevation, i.e., palatal elevation technique (PET), gave written consent for the procedure. The following steps were carried out for effective and sound delivery of the maxillary third molar in question.

Step 1: The dental chair was tipped backward so that the maxillary occlusal plane was oriented at 45-60 degrees to the floor when the patient opened his/her mouth. The height of the chair was adjusted in such a way that the patient's mouth was at a level between the patient's shoulder and elbow.

Step 2: Adequate anesthesia was secured using a combination of posterior superior alveolar nerve block and greater palatine nerve block.

Step 3: The sharp end of the No 9 Molt's Periosteal Elevator was then used to gently reflect first, the interdental papillae and then the marginal gingivae around the teeth paying special attention to reflecting the gingiva that is firmly adherent onto the distal aspect of the tooth. In the author's view, this simple maneuver was shown to prevent fracture of the maxillary tuberosity as relatively stronger forces were seen to be generated when palatal bone was used as a fulcrum which can predispose to fracture of the tuberosity if the distal gingiva around the tooth is not reflected.

**Figure 1:**
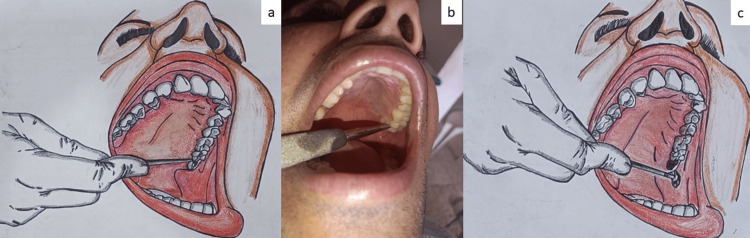
Illustration of the palatal elevation technique (PET). a, b: Application of the dental elevator from the contralateral corner of the mouth onto the palatal aspect. Note the need for good mouth opening for proper application of the elevator. The retained palatal tooth structure and the palatal bone provide a good purchase point for the luxation of the tooth. c: Delivery of the offending tooth.

Step 4: The patient was then advised to open his/her mouth wide followed by the application of the straight elevator from the contralateral angle of the mouth (Figures 1a, 1b).

Step 5: The elevator was made to engage between the palatal bone on the mesial side of the maxillary third molar and the tooth structure. The thumb on the other hand was positioned onto the occlusal surfaces of the second and third molars to make sure that while imparting luxation forces only the third molar was luxated and not the second molar. The palatal bone was used as the fulcrum, and the tooth was gently luxated out from its socket and either delivered completely out or was delivered using extraction forceps (Figure 1c). The operator was positioned at the 7 o'clock position when performing the PET for luxation of either the left or right offending maxillary third molar tooth.

**Figure 2:**
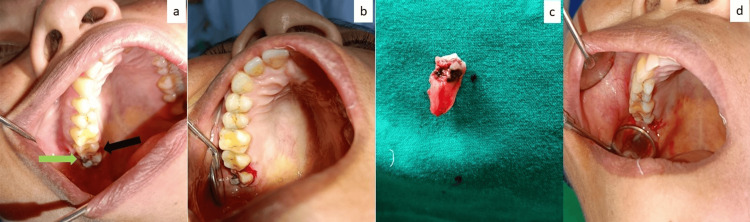
Demonstration of the palatal elevation technique (PET) in a patient. a: Photograph showing the grossly decayed maxillary right third molar with the decayed mesiobuccal portion of the tooth extending below the cementoenamel junction (green arrow). This situation precludes the usage of the dental elevator from the buccal aspect and does not allow for luxation of the tooth. The intact tooth portion on the mesial aspect of the tooth on the palatal side (black arrow) is used as the fulcrum for PET in such a situation. b: Reflection of the palatal gingiva for sound application of the dental elevator to prevent any injury or tear to the gingiva during luxation. c: Delivery of the offending tooth. d: Intra-oral view showing the sound condition of the gingiva and socket post-extraction.

The authors have utilized the PET for the extraction of more than 300 grossly carious maxillary third molars in whom tooth luxation by using the dental elevator from the buccal aspect of the offending tooth was not possible. Three cases of maxillary tuberosity fractures were noted attributed to insufficient reflection of the gingiva on the distal aspect of the tooth in all cases. No complex complications were noted including hematoma, buccal plate fracture, fracture/luxation of adjacent tooth, etc. The healing of the extraction wound in all the patients was found to be uneventful (Figures 2a-2d).

## Discussion

Intra-alveolar extraction of maxillary third molars always poses a challenge to dental practitioners owing to limited accessibility and minimal space for the correct application of dental forceps. Dental elevators facilitate the extraction of such teeth. In the traditional technique as described in the literature, the elevator always engages the space between the interdental bone and the offending tooth from the mesiobuccal aspect to use it as a fulcrum [4]. This technique is found to be effective in most situations and allows for adequate luxation and subsequent removal of the offending third molar either alone or with the help of dental forceps. Certain situations prevent the application of dental elevators from the buccal aspect of the tooth. One such situation is a grossly decayed third molar especially from the mesiobuccal aspect with destruction of the tooth substance extending below the cementoenamel junction [4]. In such a situation, the elevator will fail to engage the space between the interdental bone and the tooth resulting in inadequate or no luxation [4]. Another situation is observed in patients presenting with thick and inextensible cheeks but a good interincisal opening. In this situation, proper application of the elevator from the buccal aspect requires stretching of the cheek tissues which adds to the discomfort of the patient. Also, it becomes very challenging to achieve a good purchase for luxation of the offending third molar in such patients. In such situations, the modified technique, i.e., PET, proposed by the authors is found to be beneficial. The PET utilizes the space between the palatal interdental bone and the offending tooth from the mesial side as the fulcrum to bring about luxation and subsequent removal of the offending tooth. When the maxillary third molar is destroyed by caries from the mesiobuccal aspect and the caries extends below the cementoenamel junction (CEJ), it is observed in almost all such cases based on authors' experience that the palatal tooth structure on the mesial aspect is left intact. In the authors' view, this retained tooth structure and the strong palatal interdental bone allow for the effective application of the dental elevator from the palatal aspect to bring about intra-alveolar extraction of grossly decayed third molars with much ease and without resorting to advanced exodontia techniques of tooth removal such as performing a trans-alveolar or open extraction using bur and handpiece or chisel and mallet. Furthermore, both conical and multirooted third molars can be effectively luxated using PET in a quick manner. Another advantage of our technique is that it is simple to perform even at the hands of amateur dental professionals and requires no learning curve to master the technique. Also, patients with limited cheek extensibility but with a good interincisal opening can benefit from this novel technique.

However, there are some limitations to the use of PET. Restricted mouth opening poses a challenge in the proper application of the elevator onto the palatal side therefore limiting the usefulness of this technique in patients presenting with trismus due to oral submucous fibrosis, facial space infections, temporomandibular joint ankylosis, etc. In such situations, the traditional technique would be advantageous and is preferred over the PET. Also, in cases of buccally positioned maxillary third molars, adequate purchase cannot be created between the palatal bone and the tooth due to the buccal orientation of the tooth in the arch thereby limiting the usefulness of PET. Effective luxation of such teeth can be brought about using the traditional technique. Furthermore, no matter which technique of tooth luxation is employed, it is been observed that luxation forces on alveolar bone can cause alveolar bone fracture or cause necrosis of the bone cells lining the socket due to pressure-induced or compression-induced necrosis [6-8]. The authors carried out a thorough search of English literature and found out that such a technique of tooth luxation has not been reported in the literature so far thus deserving a mention.

Despite the judicious use of dental elevators, complications are not uncommon. The most commonly reported complications associated with the use of dental elevators for luxation of maxillary third molars from the buccal aspect include fracture of the alveolar bone, fracture of the maxillary tuberosity, fracture/luxation of the adjacent tooth, hematoma, accidental penetration of the maxillary antrum and soft tissues as well as forcing the tooth/root into the maxillary antrum [9]. With the PET, the authors have not reported any complex complications other than three cases (out of 300) of maxillary tuberosity fracture which the authors have attributed to inadequate reflection of the gingiva on the distal aspect of the tooth. This observation also points toward the usefulness of PET over the traditional technique. Despite the advantages of PET over the traditional buccal elevation technique as well as minimal reported complications, the authors are still of the opinion that a prospective study comparing the two techniques is required to substantiate our claims.

## Conclusions

The authors have modified the traditional technique of tooth elevation and named it “PET”. In the authors' view, the PET with its obvious advantages of being simple, quick to perform, easy to master and avoidance of either bur and handpiece and chisel and mallet can be thought of as a viable technique for luxation and subsequent extraction of grossly carious maxillary third molars in all those situations which do not allow for effective luxation of the offending tooth using the dental elevator from the buccal aspect. The technique can also be utilized as an alternative to buccal tooth elevation for intra-alveolar extraction of maxillary third molars which would otherwise be luxated using the elevator from the buccal aspect. However, buccally positioned third molars or patients with restricted mouth opening are limitations to sound utilization of PET. Every dental practitioner should take cognizance of this technique and add it to their armamentarium to facilitate managing buccally grossly decayed maxillary third molars in the simplest way possible without resorting to more advanced exodontia techniques of tooth removal.
